# Dcas Supports Cell Polarization and Cell-Cell Adhesion Complexes in Development

**DOI:** 10.1371/journal.pone.0012369

**Published:** 2010-08-24

**Authors:** Nadezhda Tikhmyanova, Alexei V. Tulin, Fabrice Roegiers, Erica A. Golemis

**Affiliations:** 1 Fox Chase Cancer Center, Philadelphia, Pennsylvania, United States of America; 2 Department of Biochemistry, Drexel University Medical School, Philadelphia, Pennsylvania, United States of America; University of Dayton, United States of America

## Abstract

Mammalian Cas proteins regulate cell migration, division and survival, and are often deregulated in cancer. However, the presence of four paralogous Cas family members in mammals (BCAR1/p130Cas, EFS/Sin1, NEDD9/HEF1/Cas-L, and CASS4/HEPL) has limited their analysis in development. We deleted the single Drosophila Cas gene, *Dcas*, to probe the developmental function of *Dcas*. Loss of *Dcas* had limited effect on embryonal development. However, we found that Dcas is an important modulator of the severity of the developmental phenotypes of mutations affecting integrins (*If* and *mew*) and their downstream effectors *Fak56D* or *Src42A*. Strikingly, embryonic lethal *Fak56D-Dcas* double mutant embryos had extensive cell polarity defects, including mislocalization and reduced expression of E-cadherin. Further genetic analysis established that loss of *Dcas* modified the embryonal lethal phenotypes of embryos with mutations in E-cadherin (*Shg*) or its signaling partners p120- and β-catenin (*Arm*). These results support an important role for Cas proteins in cell-cell adhesion signaling in development.

## Introduction

Cas proteins are non-catalytic scaffolding proteins that control signaling relevant to cell attachment, migration, cycle, and survival (reviewed in [1], [2], [3]). The four paralogous Cas family proteins in vertebrates include BCAR1/p130Cas [4], NEDD9/HEF1/Cas-L [5], EFS/Sin [6] and CASS4/HEPL [7], of which BCAR1 and NEDD9 have been the most intensively studied. The best established functional role for these proteins is at focal adhesions, where they interact with FAK and Src to transmit integrin-initiated signals from the extracellular matrix to downstream effectors, leading to reorganization of the actin cytoskeleton and changes in motility and invasion [8]–[15].

Overexpression of Cas proteins contributes to the development of human cancer (reviewed in [3], [16]). BCAR1 is required for Src-dependent cellular transformation of murine fibroblasts [17], and conserves with NEDD9 the ability to enhance the production of matrix metalloproteases [18], enhancing tumor cell invasion of the extracellular matrix (ECM) [19], promoting mammary tumorigenesis and lung metastasis in MMTV-HER2 and other mouse models of cancer [19], [20]. NEDD9 has been defined as a component of an intracellular signaling switch that is important for epithelial-mesenchymal transition (EMT), based on activation of its downstream effector Rac [21]. TGFβ promotes EMT during tumor cell invasion through the ECM, and tissue remodeling in development. In mammals, TGFβ regulates both transcription and proteasomal degradation of NEDD9 [22], [23]; conversely, BCAR1 and NEDD9 reciprocally bind and regulate the activity of a subset of TGFβ effectors [24], [25], [26]. BCAR1 overexpression may predict aggressive estrogen receptor-negative cancers [27], [28]. Overexpression of NEDD9 supports oncogenic signaling in malignancies of the hematopoietic system [13], [29]–[32], and has been linked to increased cellular invasive behavior in breast and colorectal cancer cell lines [18], [33], squamous cell carcinomas of the head and neck [34], and enhanced metastatic potential in glioblastomas [35], melanomas [12], and some lung cancers [36], and to cell migration and EMT induced by chemical carcinogens [37]. Conversely, a null NEDD9 genotype significantly increases the latency of tumor incidence in the MMTV-PyVmT mammary cancer model [38].

While studies of the Cas group have emphasized important roles in cancer and other pathogenic conditions, little is known of their roles in normal development. Knockout of BCAR1 in mice leads to an embryonal lethal phenotype at day 11.5–12.5, associated with marked systemic congestion and growth retardation, and disordering of actin-based structures in the heart [17]. In contrast, knockout of NEDD9 results in viable, fertile animals, with minor defects in immune system maturation [39]. The presence of 4 paralogous family members with overlapping expression profiles [7], together with the difficulty of performing detailed phenotypic analysis in early embryonal development, have made it difficult to establish the required functions of Cas proteins in mammalian development.

By contrast, there is only a single Cas family protein in Drosophila, *Dcas* (CG1212). *Dcas* is highly expressed in the embryonic nervous system at stage 16 [40], as well as in the ventral ectoderm and ventral nerve cord primordial at earlier developmental stages (stages 9–12 [41]). The importance of DCas in Drosophila has been unclear. One recent study used an existing allele with a P-element insertion in an intron within the *Dcas* coding region, and a deficiency mutation overlapping *Dcas* and 5 adjacent genes, to establish a modifier role for *Dcas* in axonal fasciculation and axon guidance [40], but did not address the question of any potential early embryonal phenotypes. Although the protein is highly conserved with mammalian family members (68% with NEDD9 and 70% with BCAR1 [40]), null mutations in *Drosophila* orthologs of some of the most important mammalian interactors of Cas proteins, such as FAK (*Fak56*) [42] produce limited phenotypes. In the present study, we have used a FRT-excision-based strategy to delete the *Dcas* locus. Upon identification of an embryonal lethal phenotype affecting 10% of maternal-zygotic null embryos, we subsequently extensively probed the genetic interactions of Dcas relevant to cell migration and EMT. This work indicated evolutionary conservation of core Cas family signaling involving FAK, Src, and integrins. Combination of mutations in *Dcas* and *Fak56* perturbed localization of polarity markers, including particularly E-cadherin (Shotgun, Shg), implying that DCas might also interact with the E-cadherin-associated cell junctional proteins. Subsequent experiments directly testing this idea identified novel and potent genetic interactions between Dcas and the cell-cell adhesion proteins Shotgun, Armadillo and p120-catenin, influencing cell polarity. These findings inform the understanding of Cas protein action both in development and in cancer progression.

## Results

### Generation and characterization of a *Dcas* null allele

**Figure 1:**
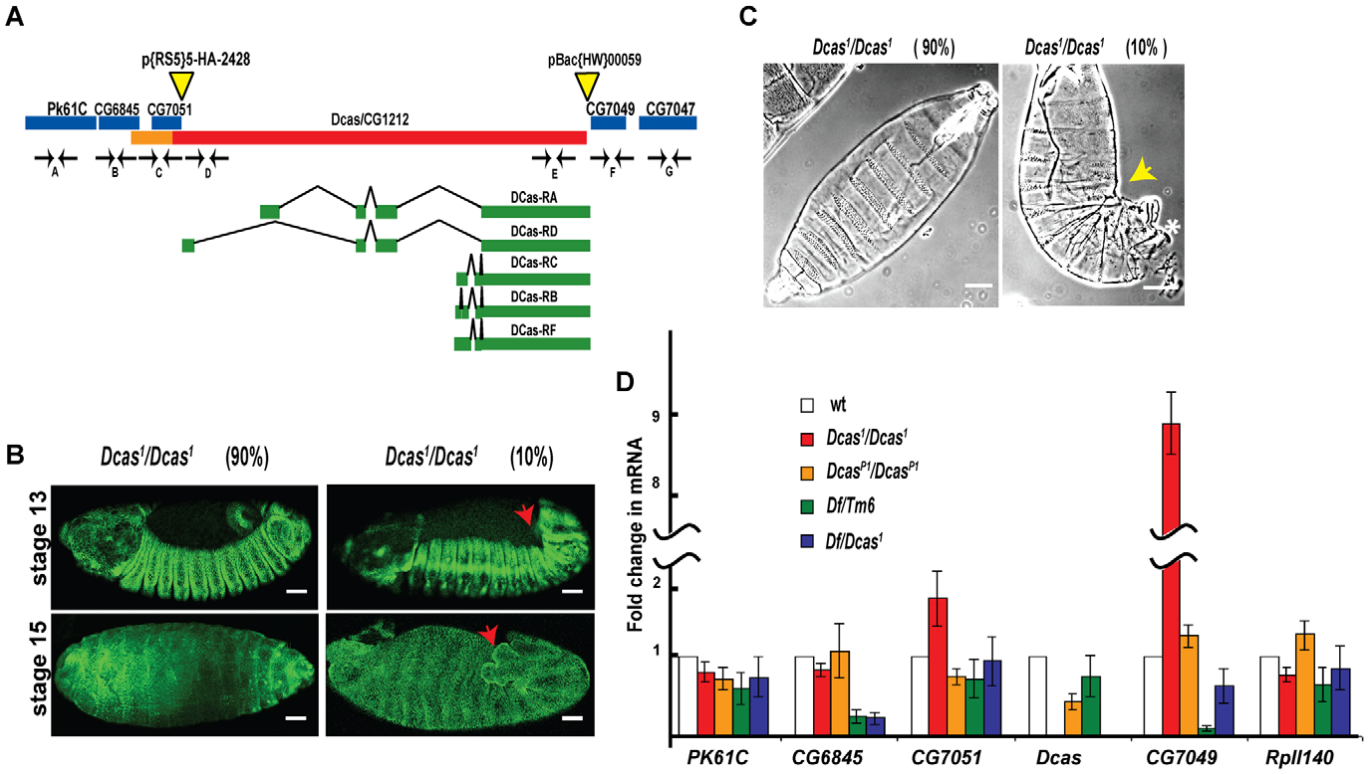
Generation of the *Dcas^1^* mutant stock. **A**. *Dcas* (CG1212) locus, with coding region (red) and promoter (orange) indicated, with alternatively spliced variant transcripts indicated below the sequence (exons shown in green, introns as black lines). Flanking genes are shown in blue. Yellow triangles mark positions of P-element insertions p{RS5}5-HA-2428 and pBac{WH}00059 used to make the *Dcas^1^* mutant. Position of primer pairs used in quantitative RT-PCR to confirm deletion of the Dcas gene (D, E) but not flanking genes (A, B, C, F, G) are indicated. **B**. Dorsal views of stage 13 and 16 *Dcas^1^/Dcas^1^* embryos stained with Fas3, indicating phenotypes of 90% (left panel) and 10% (right panel) of mutant flies. The embryos are oriented anterior to the left. Red arrow indicates characteristic “fishtail” at posterior in the 10% of embryos with DC and GBR retraction defects. **C**. Cuticle preparations of *Dcas^1^/Dcas^1^* mutant embryos; yellow arrow indicates DC and GBR defects, * indicates hole in posterior dorsal cuticle. Scale bar, 40 µm. **D**. Graph representing change in mRNA levels for indicated genes as measured by qRT-PCR analysis of cDNA prepared from wild type (white), *Dcas^1^/Dcas^1^* (red) and *Dcas^P1^/Dcas^P1^* (orange), Df(3L)Exel6083/+(green) and Df(3L)Exel6083/*Dcas^1^* (blue). Bars represent standard error.

To study *Dcas* function in Drosophila development, we used a modification of FRT-excision technology [43]. A FRT-containing P-element upstream of the *Dcas* gene was provided by a P-element located within 50 bp of the start of the Dcas open reading frame (ORF). A downstream transposon was provided by a Pbac located between the end of the *Dcas* coding sequences and the assigned start codon of the CG7049 ORF. Using this technique, we generated a precise excision of the complete *Dcas* ORF on chromosome 3 (Fig. 1A). The resulting allele, which we call *Dcas^1^*, contains a deletion spanning the first through final coding exons of the *Dcas* gene, but retains the *Dcas* promoter region and flanking genes, as confirmed by extensive quantitative PCR using probes directed against the DNA of *Dcas* and flanking genes (results not shown). Homozygous *Dcas^1^/Dcas^1^* mutants produce fertile progeny and can be maintained as a stable *Dcas* null strain.

To exclude the possiblity of secondary mutations contributing to any observed Dcas deletion-associated phenotypes, we used a number of discrete approaches to separately test the *Dcas^1^* strain. First, we crossed *Dcas^1^/Dcas^1^* stock to a stock containing the small Df(3L)Exel6083 deletion, which removes Dcas as well as Pk61c, CG6845, and CG7049 (Fig. 1A), to allow analysis of the phenotypes of *Dcas^1^/Dcas^1^* versus *Dcas^1^*/Df(3L)Exel6083 flies. Second, we crossed the *Dcas^1^/Dcas^1^* stock with a previously described *Dcas^P1^* hypomorphic allele [40] which has a GAL4-containing P-element inserted in the *Dcas* promoter, resulting in limited *Dcas* transcript levels, then analyzed *Dcas^1^/Dcas^P1^* flies. Third, in *Dcas^P1^/Dcas^P1^* flies, we also introduced the *DCas* expressing a GAL4-activated UAS promoter fusion, a UAS-GFP-Dcas transgenic allele (as described in [40]), and assessed the *Dcas^P1^*, UAS-GFP-Dcas/*Dcas^P1^*, UAS-GFP-Dcas and *Dcas^P1^*, UAS-GFP-Dcas/*Dcas^1^* phenotypes. Fourth, we assessed the mRNA expression of *Dcas* and flanking genes in the *Dcas^1^/Dcas^1^* and other mutant backgrounds.

**Figure 2:**
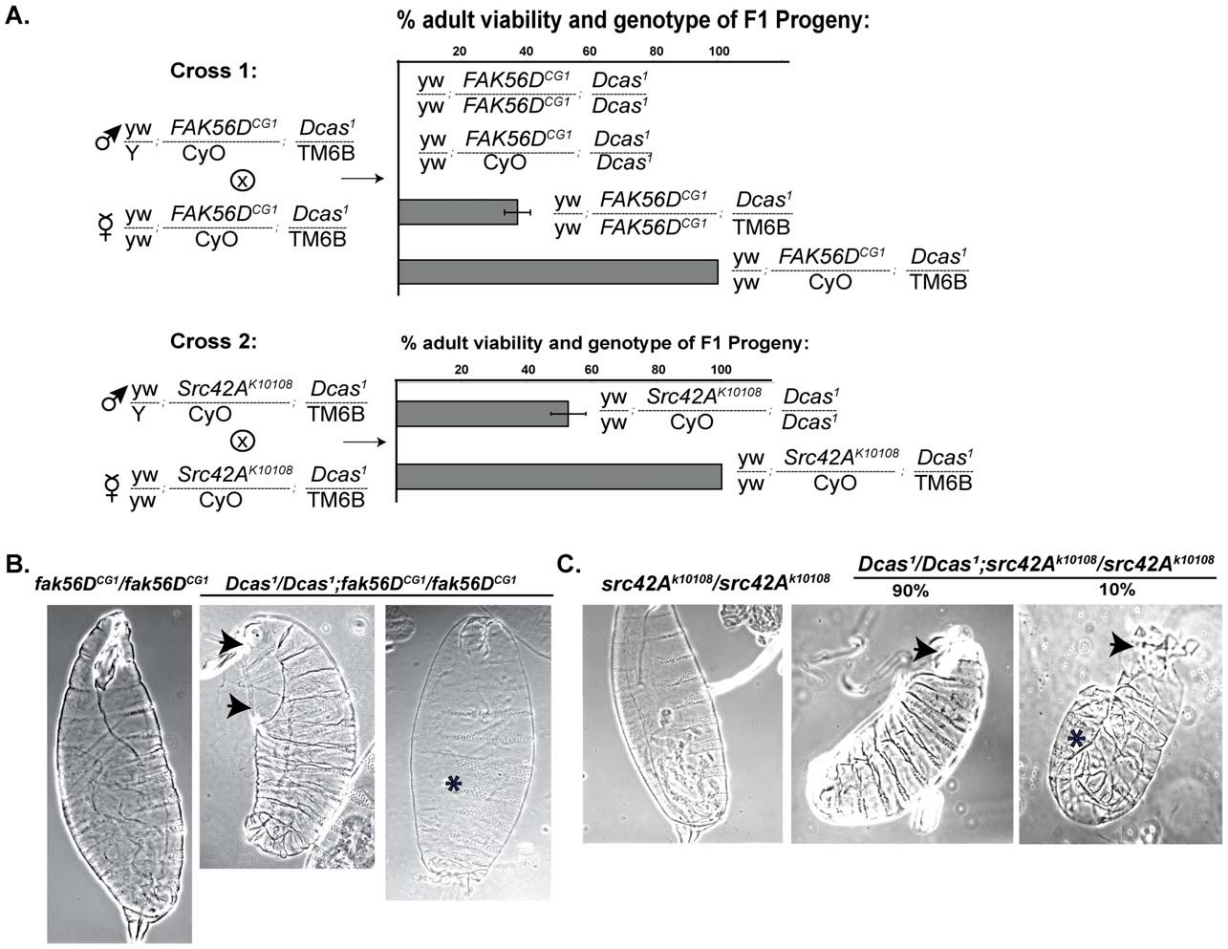
Genetic interactions of *Dcas* with *Fak56D* and *Src42A*. **A**. Represent examples of a genetic cross of two double heterozygous parents (*Fak56D^CG1^* and *Dcas^1^* in Cross 1 or *Src^K10108^* and *Dcas^1^* in Cross 2) to allow analysis of the viability of resulting progeny. Each row in the graph represents percentage of viable progeny of indicated genotype. Total number of expected progeny was calculated from the number of phenotypically viable double balanced adult heterozygotes *Fak56^CG1^/CyO*; *Dcas^1^*/TM6B, *Ubx*, *y+* in Cross 1 and *Src^K10108^/CyO*; *Dcas^1^*/TM6B, *Ubx*, *y+* in Cross 2, which we considered 100% viable. The percentage of viability for the remaining progeny was calculated in agreement with Mendel's law of independent assortment for two alleles. **B**. Cuticle preparations of stage 16 *Fak56D^CG1^/Fak56D^CG1^* and *Fak56D^CG1^/Fak56D^CG1^;Dcas^1^/Dcas^1^* embryos transitioning to 1^st^ instar larvae, viewed laterally, ventral side to right. Arrows indicate holes in head and dorsal cuticle, * indicates missing and/or fused denticle belts. **C** Cuticle preparations of stage 16 *Src42A^k10108^/Src42A^k10108^* and *Src42A^k10108^/Src42A^k10108^;Dcas^1^/Dcas^1^* embryos. Arrows indicates holes in head; * indicates GBR defect associated with incomplete DC.

While viable and fertile, the *Dcas* null stock yielded a a very weak lethal phenotype in which 10% of *Dcas^1^/Dcas^1^* embryos did not hatch but instead developed a “kink” at stage 13, and arrested at stage 15–16 of embryonic development (Table 1, Fig. 1B). These embryos had germ band retraction (GBR) and dorsal closure (DC) defects [44], including an irregular leading edge of migrating cells (not shown); and typically had embryonal curvature and a posterior opening in the dorsal cuticle (Fig. 1C, Fig. 2). Similar GBR and DC phenotypes were seen in 6% of *Dcas^1^*/Df(3L)Exel6083 embryos and 1% of the *Dcas^1^/Dcas^P1^* embryos, as were similar rates of overall lethality. No lethality was observed in *Dcas^1^*/+ embryos (n = 613). Expression of GFP-Dcas in *Dcas^P1^*, UAS-GFP-Dcas/*Dcas^P1^*, UAS-GFP-Dcas and *Dcas^P1^*, UAS-GFP-Dcas/*Dcas^1^* embryos completely rescued embryonic GBR and DC defects observed in *Dcas^P1^/Dcas^1^* and *Dcas^1^/Dcas^1^* stocks (not shown).

**Table 1:** Synthetic lethal interactions involving *Dcas^1^* and genes of the integrin signaling network.

Genotype of mutant progeny	Viability (+/−SD) (%)	Total (n)
*Dcas^1^/Dcas^1^*	88 (+/−3)	504
*fak56D^CG1^/*CyO; *Dcas^1^/Dcas^1^*	0	466
*fak56D^CG1^/fak56D^CG1^*; *Dcas^1^/Dcas^1^*	0	
*fak56D^CG1^/fak56D^CG1^*; *Dcas^1^*/TM6B	38 (+/−3)	
*src42A^k10108^*/CyO; *Dcas^1^/Dcas^1^*	53 (+/−5)	483
*src42A^miri^*/CyO; *Dcas^1^/Dcas^1^*	50 (+/−7)	652
*src42A^E1^*/CyO; *Dcas^1^/Dcas^1^*	48 (+/−5)	650
*src42A^JP45^*/CyO; *Dcas^1^/Dcas^1^*	29 (+/−5)	751
*mys^1^*/FM7i-GFP, *B;Dcas^1^/Dcas^1^*	24 (+/−1)	510
*If^B2^*/FM7i-GFP, *B;Dcas^1^/Dcas^1^*	56 (+/−10)	436
*If^3^/If^3^;Dcas^1^/Dcas^1^*	27 (+/−17)	747
*If^3^/If^3^;Dcas^1^*/TM6B	25 (+/−2)	
*If^3^*/FM7i-GFP, *B;Dcas^1^/Dcas^1^*	38 (+/−4)	
*mew^EY09631^/mew^EY09631^;Dcas^1^/Dcas^1^*	0	948
*mew^EY09631^*/FM7i-GFP, *B*; *Dcas^1^/Dcas^1^*	8 (+/−1)	
*mew^EY09631^/mew^EY09631^;Dcas^1^*/TM6B	107 (+/−22)	
*mew^G0429^*/FM7i-GFP, *B;Dcas1/Dcas1*	29 (+/−11)	388

**Figure 3:**
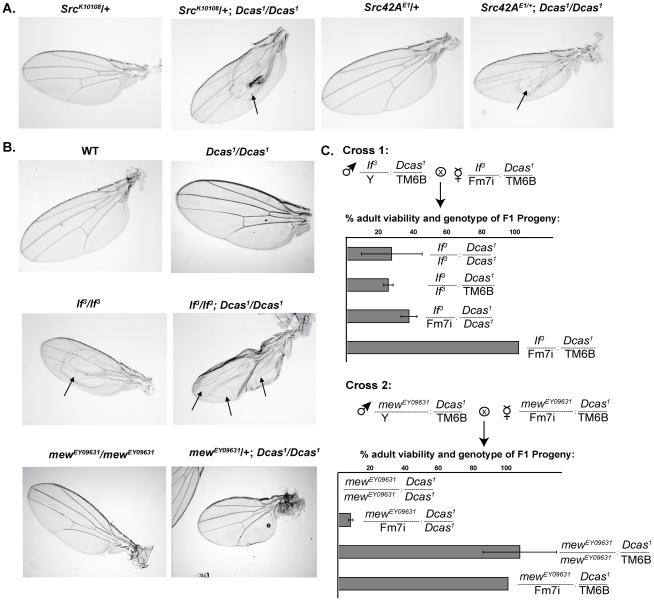
Absence of *Dcas* induces wing defects in *Src42A*, *If*, *and mew*-deficient flies. **A**. *Dcas^1^/Dcas^1^* genotype induces a blister (arrows) phenotype in *Src42A* heterozygous mutant flies. **B**. Arrows point to typical wing blisters in wings of the flies of the indicated genotypes. **C**. Representative genetic cross of two double heterozygous parents (*If^3^* and *Dcas^1^*; mew^EY09631^ and *Dcas^1^*) to allow analysis of the viability of resulting progeny. Each row in the graph represents percentage of viable progeny of indicated genotype. Only female progeny were analyzed.

For data shown, the parental crosses were performed as described in Methods and shown in Figures 2A and 3C. The viable adult progeny of indicated genotypes was collected and compared to phenotypically normal double heterozygous siblings (i.e. *Fak56^CG1^/CyO*; *Dcas^1^*/TM6B, *Ubx*, *y+*) in each of 3 independent experiments.

Analysis of cDNA prepared from *Dcas^1^/Dcas^1^* stocks indicated complete absence of *Dcas* transcript. *Dcas^P1^/Dcas^P1^ mutants* had significantly reduced but still detectable levels of the Dcas transcript (Figure 1D). While the *Dcas^1^/Dcas^1^* stock had somewhat elevated expression of the adjacent CG7049 locus (which is predicted to encode a protein, but has no described phenotype or known function), the *Dcas^1^/Df(3L)Exel6083* and *Dcas^P1^/Dcas^P1^* stocks did not: indeed, gene expression of CG7049 was diminished in Df(3L)Exel6083/+ stock. Together, these expression results argue against variation in CG7049 expression as contributing to the observed GBR/DC phenotype (Figure 1D), and suggest the minimal phenotype observed with the *DCasP^1^* allele reflect the fact that this strain reduces (to 43% of wt) but does not eliminate *DCas* mRNA expression.

### Synthetic lethality of *Dcas^1^* with *FAK56* and *Src42A* mutations, and modifier interactions between *Dcas* and integrins

The best defined signaling partners of Cas proteins in mammals are components of the integrin signaling network. For instance, in mammals, interactions of the Cas proteins with FAK, Src, and integrins are critical for cell migration [1], [16]. We hypothesized that the weak *Dcas^1^* phenotypes might be exacerbated by additional targeting of the Drosophila orthologs of these genes. The Drosophila FAK ortholog, *Fak56D*, is not essential for viability or fertility and a null mutation, *Fak56^CG1^*, has no gross phenotypes associated with cell migration [45], although homozygous mutations in *Fak56D* have been associated with morphogenesis of the optic stalk in second and third instar larvae [42]. Drosophila have two Src-related genes, *Src42A* and *Src64*, which have redundant function in GBR and DC (with double mutants having phenotypes similar to those seen in 10% of *Dcas^1^/Dcas^1^* mutants, [46]), and other developmental processes [47]. Homozygous null alleles in *Src42A* have a high frequency of death before hatching or as first instar larvae, although some adult escapers of the hypomorphic allele *Src42A^JP45^* have mild dorsal cleft phenotypes [46].

We first analyzed the genetic interactions of *Dcas^1^* with *Fak56^CG1^*. We created a double-balanced stock which carried both *Dcas^1^* and *FAK56D^CG1^* mutations. *Dcas^1^/Dcas^1^* in combination with either heterozygous or homozygous *Fak56^CG1^* yielded no viable adult offspring (Table 1, Figure 2A). The *Fak56^CG1^/Fak56^CG1^*; *Dcas^1^/+* genotype also significantly reduced the viability of adults. Analysis of the *Fak56^CG1^/Fak56^CG1^*; *Dcas^1^/Dcas^1^* lethal phenotype indicated that most (95%) of the embryos did not hatch. The few escapers survived to pupal stages, but did not emerge. We then crossed double mutants to a stock with a green compound balancer *CyO-TM3-GFP*, which constitutively expresses GFP from the *Hsp70* promoter during all developmental stages, and selected *Fak56^CG1^*, Dcas^1^/CyO-TM3-GFP embryos, to separate double homo- and heterozygotes for analysis of the cuticles of double homozygotes (Figs 2A). In contrast to embryos with either the *Dcas^1^* or *Fak56^CG1^* homozygotes, only 5% of *Fak56^CG1^/Fak56^CG1^*;*Dcas^1^/Dcas^1^* mutants produced cuticles (Fig. 2B), and almost all observed cuticles were marked by dorsal and/or ventral holes, indicating dorsal closure defects. Additionally, *Fak56^CG1^/Fak56^CG1^*; *Dcas^1^/Dcas^1^* cuticles had fused or missing (not shown) denticle belts, phenotypes never observed in cuticles of *Fak56^CG1^* homozygotes alone.

*Src42A^k10108^* is a mild hypomorphic allele of Src42A: *Src42A^k10108^/Src42A^k10108^* homozygous embryos hatch, but die as first instar larvae from defects in tail morphology, head involution, and tracheal necrosis [48]. However, we observed synthetic lethality in *Dcas^1^/Dcas^1^*; *Src42A^k10108^*/CyO adult flies (Table 1, Figure 2A, C) and *Dcas^1^/Dcas^1^*; *Src42A^k10108^*/*Src42A^k10108^* embryos, as fewer than 46% of these double mutant embryos hatched (data not shown). Cuticles were assessed by a strategy similar to that described for *DCas^1^*×*Fak56D^CG1^* mutants. 90% of the cuticles of *Src42A^k10108^*/*Src42A^k10108^*; *Dcas^1^/Dcas^1^* embryos had holes in or absence of the head cuticle, and 10% of *Dcas^1^/Dcas^1^*; *Src42A^k10108^*/*Src42A^k10108^* cuticles had additional GBR defects. Similar phenotypes were obtained crossing *Dcas^1^/Dcas^1^* to two additional Src42A mutant strains including the mild allele *Src42A^miri^* and the strong allele *Src42A^E1^* (Table 1 and not shown).

We confirmed the synthetic lethal interactions between *DCas* and *Src42A* and *Fak56D*, by in each case also assessing the phenotype of the *Src* and *Fak* alleles with *Dcas^1^/Df(3L)Exel6083*, in order to exclude the influence of potential secondary mutations in the *Dcas^1^/Dcas^1^* stock. *Fak56^CG1^*/CyO; *Dcas^1^*/Df(3L)Exel6083 adults did not emerge, indicating complete lethality. *Src42A^k10108^*/CyO; *Dcas^1^*/Df(3L)Exel6083 and *Src42A^E1^*/CyO; *Dcas^1^*/Df(3L)Exel6083 were semi-viable, emerging at approximately 50% of the rate of phenotypically normal adult siblings from the same cross (results not shown). Interestingly, the same cross to a double balanced *Dcas^P1^* allele did not result in the same substantial decrease in the numbers of *Src42A^E1^*/CyO; *Dcas^1^/Dcas^P1^* progeny, implying that moderate expression of *Dcas* is sufficient to support the survival of *Src42A^E1^* mutants.

Interestingly, the low percentage of *Src42A^k10108^*/*+*; *Dcas^1^/Dcas^1^ and Src42A^E1^*/*+*; *Dcas^1^/Dcas^1^* adult escapers manifested wing blistering defects similar to those seen with mutants in integrin subunits [49] (Figure 3A). These data implied that simultaneous reduction in *Dcas* and *Src42A* function combined to impact an important integrin-dependent effector pathway. Based on these results, we also assessed whether *Dcas* interacted genetically with Drosophila orthologs of integrin α (*mew^EY09631^* and *mew^G0429^*; *If^B2^* and *If^3^*) and β (*mys^1^*) subunits, which are upstream activators of SRC and FAK. Double balanced stocks of integrin mutants and *Dcas^1^* were crossed to make double heterozygous stocks of each mutant in combination with Dcas, and analyzed for adult viability and visible phenotypes (Table 1).

Loss of Dcas in *If* mutants only moderately affected viability of adult flies: 27% of *If^3^/If^3^*; *Dcas^1^/Dcas^1^* and 56% of *If^B2^/+*; *Dcas^1^/Dcas^1^* emerge as adults (Figure 3B). However, the characteristic wing blistering defects of the *If* integrin α mutants [49] were significantly exacerbated in all viable progeny of double homozygous *If^3^/If^3^*; *Dcas^1^/Dcas^1^* (Table 1, Figure 3B). Further, loss of a single or both copies of Dcas in combination with mew^EY09631^ (a viable weak hypomorphic allele) caused a dramatic reduction in the viability of adult mew^EY09631^ homozygotes (Table 1). Moreover, 2% of *mew^EY09631^/+*; *Dcas^1^/Dcas^1^* flies had wing blisters and smaller, more rounded wings (Figure 3B). These interactions were enhanced using a lethal allele of mew (mew^G0429^), with viability of mew^G0429^/*+*; *Dcas^1^/Dcas^1^* significantly reduced (Table 1). Finally, loss of *Dcas* significantly lowered the percentage of viable *Dcas^1^/Dcas^1^*; *mys^1^*/*+* adults (Table 1), although no wing phenotypes were observed (not shown).

### *Dcas* interacts with *Fak56D* to influence cell polarity and cytoskeleton

**Figure 4:**
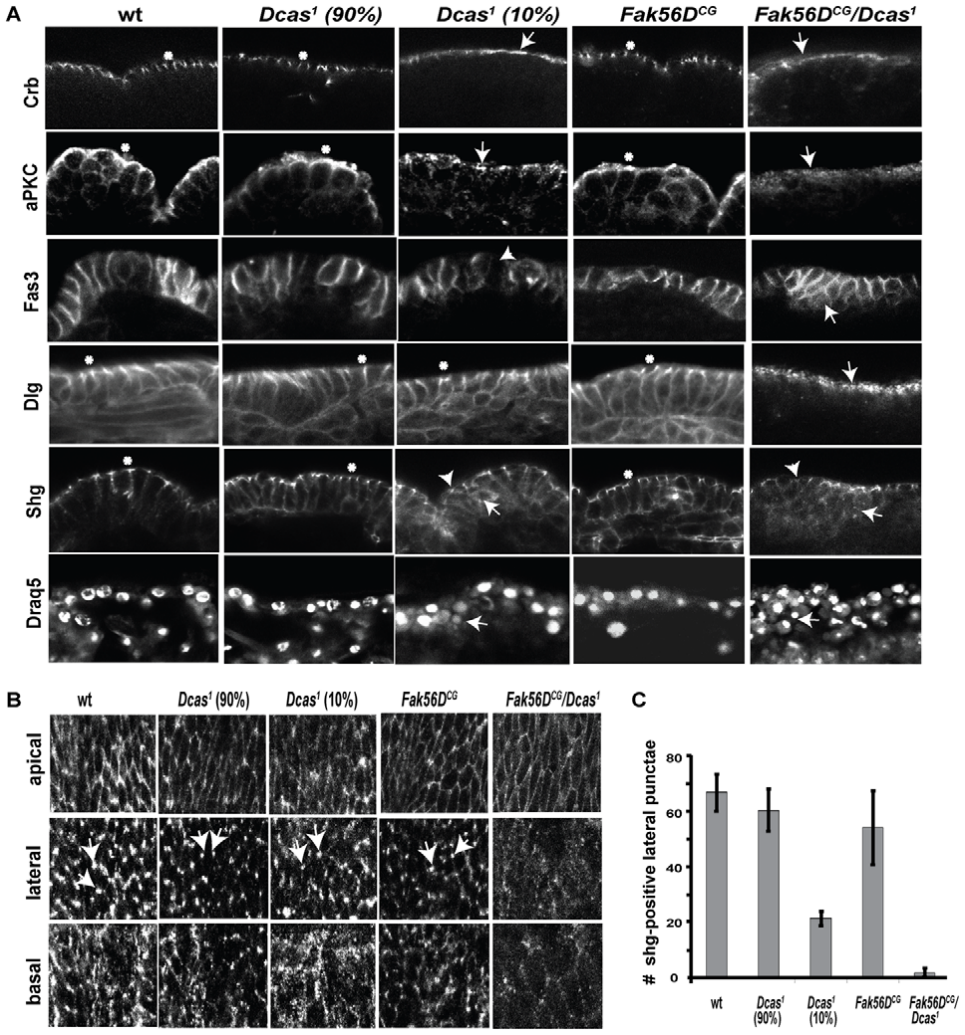
Cell polarity consequences of mutations in Dcas and Fak56. **A**. Immunofluorescence of epithelial cells of stage 15 embryos with indicated homozygous mutant genotypes, visualized with antibodies to Crb, aPKC, Fas3, Dlg, and Shg, as indicated. Arrows and arrowheads indicate defects in the localization of apico-basal polarity determinants or morphology changes in mutants, while asterisks indicate wild type appearance for each marker. In Crb panels, arrows point to a apically diffused localization of Crb in *Dcas^1^* (10%) embryos, and reduced and diffuse localization of Crb in homozygotic *Dcas^1^/Fak56^CG1^* embryos. In aPKC panels, arrows indicate abnormally punctate localization of Crb in *Dcas^1^* (10%) embryos and diminished and diffuse localization of Crb in homozygotic *Dcas^1^/Fak56^CG1^*. The abnormally punctate and apical localization of Dlg is indicated with an arrow in homozygous *Dcas^1^/Fak56^CG1^* embryos. In *Shg* panels, arrowheads point to cell junctions with reduced visibility of lateral punctae, and arrows indicate the increased cytoplasmic localization of Shg in *Dcas^1^* (10%) and *Dcas^1^/Fak56^CG1^* embryos. In Fas3, arrowhead points to a rounded cell within the epithelial layer in a *Dcas^1^* (10%) embryo stained with Fas3. Multilayering of cells in the epithelium of *Dcas^1^/Fak56^CG1^* and/or *Dcas^1^* (10%) embryos is apparent in embryos stained with Fas3 or Draq5. **B**. Immunofluorescence with antibody to Shg visualizing apical, lateral, and basal z-series of stage 15 embryonal epithelial cells from flies with indicated genotypes. Z-sections were taken starting from the apical surface, with increments of 0.1 µm. Lateral images shown here reflect the 5^th^ section (0.5 µm) and basal reflects the 10^th^ section (1 µm) down from the apical surface. Lateral punctae are marked with arrows. **C**. Quantification of punctate E-cadherin-positive lateral junctions in flies of indicated homozygotic genotypes, per 35 µm^2^. More than 6 embryos in 3 independent experiments were analyzed.

Based on the defined biology of mammalian Cas proteins (reviewed in [50]), the defects seen with *Dcas* and *Fak56* mutant flies may reflect defects in cellular morphology (e.g., attachment and polarization) that inhibit appropriate migration during development. To begin to explore these mechanisms, we assessed the localization of markers of apical and basolateral polarity in flies with mutations in *Dcas* and *Fak56*. We compared localization of a set of polarity markers in embryos undergoing DC in mutants and wild type homozygotes and heterozygotes. Localization of polarity markers in heterozygous mutants was in all cases comparable to wild type (not shown). (*Crumbs*, CRB1) and aPKC localize to the subapical region/marginal zone in wild type embryos. For Crb, this localization was diminished in the 10% of phenotypically affected *Dcas^1^/Dcas^1^* mutant embryos, while in *Dcas^1^/Dcas^1^;* *Fak56^CG1^/Fak56^CG1^ Dcas^1^/Dcas^1^* embryos, Crb staining was generally reduced and diffuse in the cytoplasm (Fig 4A). aPKC staining was abnormally punctate specifically in the 10% of *Dcas^1^/Dcas^1^*embryos which had discernible GBR defects. Staining intensity of aPKC was both generally reduced and more cytoplasmically diffuse in *Dcas^1^/Dcas^1^;* *Fak56^CG1^/Fak56^CG1^* embryos (Fig 4A).

The septate junction markers Fas3 (*fasciclin 3*) and Dlg (*Discs large*), and an adherens junction marker, Shg (*shotgun*, E-cadherin), localize to the basolateral cell surface of epithelial cells. Fas3 expression and localization were unaffected in *Dcas^1^/Dcas^1^*; *Fak56^CG1^/Fak56^CG1^* embryos beginning DC, although staining suggested a multi-layering of cells that was also indicated by the nuclear staining pattern obtained with the DNA label DRAQ5. Dlg staining patterns become more punctate and apical in *Dcas^1^/Dcas^1^;* *Fak56^CG1^/Fak56^CG1^* embryos. Interestingly, Shg staining was markedly altered (Figs 4A–C) in embryos lacking *Dcas*, *Fak56*, or both. Shg staining in double mutant embryos was more cytoplasmic and diffuse compared to same stage embryos of other genotypes, with particular reduction of intense staining in the apical and lateral compartments. Detailed analysis of the intracellular distribution of Shg (Figs 4B, C) revealed significant reduction in the lateral punctate Shg staining in *Dcas^1^/Dcas^1^;* *Fak56^CG1^/Fak56^CG1^* embryos. Although immunofluorescence analysis indicated Shg expression was maintained overall in Dcas null embryos, the 10% of embryos with GBR/DC defects had evidence of mislocalized Shg, with greater accumulation in a disorganized pool of Shg at the lateral and basal cell surface.

### Dcas and Fak56 negatively regulate *shg*/E-cadherin protein localization in Drosophila embryos

**Figure 5:**
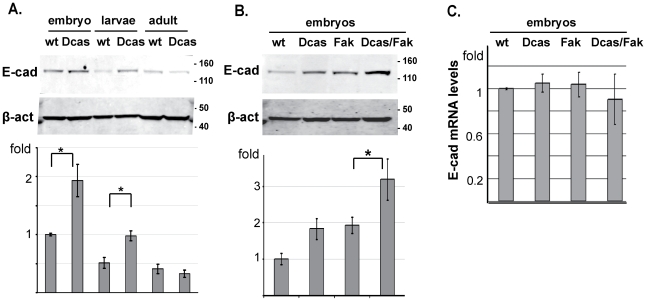
Dcas negatively regulates expression of E-cadherin. **A**. Western analysis of lysates prepared from wt or *Dcas^1^/Dcas^1^* (*Dcas*) stage 13–16 embryos, 1^st^–2^nd^ instar larvae, or adult flies with antibody to DE-cadherin. β-actin was used as loading control. Graph below compares E-cadherin normalized β-actin based on results of 3 independent experiments; *, P = 0.003 **B**. Western analysis of lysates from wt, *Dcas^1^/Dcas^1^* (*Dcas*), FAK56D^CG1^/FAK56D^CG1^ (*fak*) and Dcas^1^/Dcas^1^; Fak56D^CG1^/Fak56D^CG1^ (*Dcas/fak*). Graph as in **A**, *, P = 0.005. **C**. Expression levels of E-cadherin mRNA in stage 13–16 embryos of the indicated genotypes, as established by RT-PCR. Differences are not statistically significant.

The loss of *Shg* from the adherens junctional complex might reflect defects in localization of the protein, or reduced Shg expression. To discriminate these possibilities, we analyzed extracts made from Drosophila embryos (stage 13–16), 2^nd^–3^rd^ instar larvae, and adults (Fig. 5A). Quantitive Western blot analysis indicated that *Dcas^1^/Dcas^1^* embryos or larvae contain 2-fold higher levels of E-cadherin/Shg compared to WT, although no differences were seen in adult flies (Fig. 5A, graph). We next compared E-cadherin/Shg expression in *Dcas^1^/Dcas^1^*, *Fak56^CG1^/Fak56^CG1^* or *Dcas^1^/Dcas^1^*;*Fak56^CG1^/Fak56^CG1^* embryos. A *Fak56^CG1^/Fak56^CG1^* genotype elevated E-cadherin protein levels to the same extent as *Dcas^1^/Dcas^1^*, while the double mutant had 3.1-fold more protein relative to wild type levels (Fig. 5B). E-cadherin transcription levels were not affected in Dcas and/or FAK mutants. The simplest interpretation of these results is that Shg does not effectively localize to lateral junctions in the absence of *Dcas* and *Fak56*. This defect initiates compensatory signals that modestly upregulate Shg at the level of translational control or protein stability, but this Shg remains trapped in the basal cytoplasmic cellular compartment.

Interestingly, Crb has been reported to support Shg localization to adherens junctions [51]. The fact that the localization of Crb, Dlg, and Shg, but not Fas3, was strongly affected in the assessed double mutants indicated that *Dcas* and *Fak56* did not ubiquitously affect localization controls. Further, in Drosophila, the Src orthologs genetically and functionally interact with *shg*, and phosphorylate the Shg partner protein *armadillo* (*arm*, β-catenin) promoting its degradation [47], [52]. As our data indicated *Dcas* both interacted with *FAK56D* and *Src42A* and resulted in mislocalized *shg*, these results together suggested close interactions between Dcas and Shg function.

### *Dcas* genetically interacts with *Shg*

**Figure 6:**
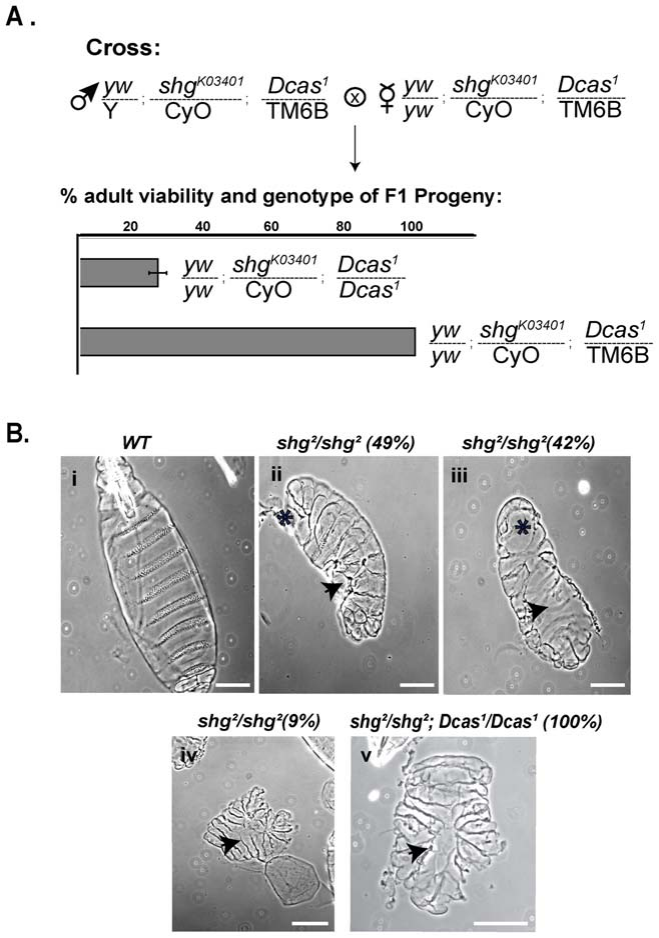
Genetic interactions of Dcas with *shg*. **A**. Representative genetic cross of two double heterozygous parents (*shg^K03401^* and *Dcas^1^*) to allow analysis of the viability of resulting progeny. Each row in the graph represents percentage of viable progeny of indicated genotype. **B**. Cuticle preparations of WT, *shg^2^/shg^2^* and *Dcas^1^/Dcas^1^*;*shg^2^/shg^2^* stage 16 embryos, viewed ventrally (panels i and iii), laterally (panel ii) and dorsally (panels iv–v). * indicates defects in head and ventral cuticle formation, respectively, arrows point to holes in ventral and dorsal cuticle. Genotypes and percentages of cuticles with indicated phenotypes are marked on top. Scale bar, 100 µm.

**Figure 7:**
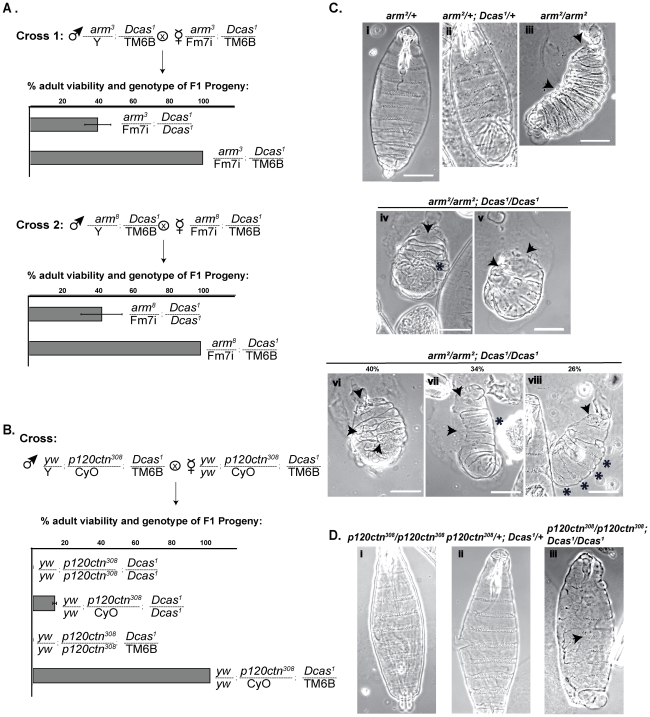
Genetic interactions of Dcas with *arm*, *shg* and *p120ctn*. **A**, **B**. Representative genetic cross of two double heterozygous parents (*arm^3^* and *Dcas^1^*, *arm^8^* and *Dcas^1^*, *p120ctn^308^ and Dcas^1^*) to allow analysis of the viability of resulting progeny. Each row in the graph represents percentage of viable progeny of indicated genotype. **C**. Cuticle preparations of stage 16 embryos of the indicated genotypes. Panels iii and v–viii, lateral view; panels i, ii, and iv, ventral view. Arrows indicate holes in head and/or dorsal cuticle, and GBR defect; * indicates fused denticles in panel iv, lack of ventral denticles in panel vii, and well-separated ventral denticle belts in panel viii. Percentages of embryos with fused, absent and well-separated denticles are shown above in panels iv-vi. **D**. Cuticle preparations of stage 16 embryos of the indicated genotypes. Arrow indicates fused ventral denticle belts.

We therefore next assessed genetic interactions between the *Dcas^1^* allele and loss-of-function alleles of *shotgun* (*shg^2^*, encoding an unstable protein that is prone to degradation [53]; *shg^E17B^*, a genetic null mutation producing a defective DE-cadherin; and *shg^K03401^*, produced by a P-element interruption of gene transcription) [54], and its functional partners *armadillo* (*arm^2^*, *arm^3^*, *arm^8^*,) and *p120catenin* (*p120ctn^308^*). Neither heterozygotic alleles of *shg^2^*, *shg^E17B^ p120ctn^308^* and *arm^2^*, nor double heterozygotes of *Dcas^1^* and any of these genes produced visible phenotypes or reduced the emergence of adult flies (Figure 6 and Table 2). However, *Dcas^1^/Dcas^1^* in combination with heterozygous *shg^K03401^*, *arm^3^* or *arm^8^*, or homozygous *p120ctn^308^*, severely reduced viable adult progeny, as did combination of *Dcas^1^/+* with *p120ctn^308^/p120ctn^308^* (Figures 6A, 7A, 7B, and Table 2).

**Table 2:** Synthetic lethal interactions involving *Dcas^1^* and alleles of shg, arm, and p120ctn.

Genotype of mutant progeny	Viability (+/−SD) (%)	Total (n)
*shg^2^*/CyO ; *Dcas^1^/Dcas^1^*	0	1057
*shg^E17B^*/CyO; *Dcas^1^/Dcas^1^*	0	658
*shg^K03401^*/CyO; *Dcas^1^/Dcas^1^*	26 (+/−3)	511
*arm^2^/*FM7i-GFP, *B*; *Dcas^1^/Dcas^1^*	0	526
*arm^3^*/FM7i-GFP, *B*; *Dcas^1^/Dcas^1^*	39 (+/−7)	463
*arm^8^*/FM7i-GFP, *B*; *Dcas^1^/Dcas^1^*	42 (+/−12)	573
*p120ctn^308^*/p120ctn^308^ ; *Dcas^1^/Dcas^1^*	0	648
*p120ctn^308^*/CyO ; *Dcas^1^/Dcas^1^*	12 (+/−1)	
*p120ctn^308^/p120ctn^308^*; *Dcas^1^*/TM6B	0	

For data shown, the parental crosses were performed as described in Methods and shown in Figures 5A, 6A and 6B. The viable progeny of indicated genotypes was collected and compared to phenotypically normal double heterozygous siblings, (i.e. *p120ctn^308^/CyO*; *Dcas^1^*/TM6B, *Ubx*, *y+*) in each of 3 independent experiments.

**Abbreviations list.**

Dcas – Drosophila p130Cas.

FAK – focal adhesion kinase.

DC – dorsal closure.

GBR – germ band retraction.

*shg* is important for morphogenesis of the head and ventral epithelium [53]. Although homozygous *shg^2^* embryos complete DC due to abundant maternal contribution, some *shg^2^*/*shg^2^* embryos may have small irregularities of the leading edge [53]. However, no *shg^2^*/*shg^2^* embryos progress to 1^st^ instar larvae because of moderate (49%, Fig. 6B, panels i versus ii) to severe (42% and 9%, Fig. 6B, panels iii and iv) defects in the embryonic head and ventral cuticle. By contrast, none of *shg^2^*/*shg^2^*; *Dcas^1^/Dcas^1^* embryos hatched; rather, *shg^2^*/*shg^2^*; *Dcas^1^/Dcas^1^* double homozygotes arrested in late embryogenesis. Although most of the embryos formed at least partial cuticles, absence of Dcas significantly enhanced *shg^2^*/*shg^2^* cuticle defects, with all *shg^2^*/*shg^2^*; *Dcas^1^/Dcas^1^* embryos showing severe defects that essentially eliminated head and ventral cuticle, while incomplete DC was reflected by the presence of holes in the dorsal cuticle (Fig. 6B, panel v).

To confirm specificity of the genetic interaction between *Dcas* and *Shg*, we subsequently crossed *Dcas^1^* with two other alleles of Shg, *shg^E17B^* and *shg^K03401^*. Both alleles are embryonal lethal at embryonal stage 16, with embryos failing to produce head and ventral cuticle [53], [55]. Double mutants *shg^E17B^*/*shg^E17B^*; *Dcas^1^/Dcas^1^* and *shg^K03401^*/*shg^K03401^*; *Dcas^1^/Dcas^1^* were lethal or semi-lethal and had similarly enhanced cuticle defects, as observed with *shg^2^*/*shg^2^*; *Dcas^1^/Dcas^1^*, indicating the genetic interaction observed was not allele-specific.

### *Dcas* interacts with *arm* and *p120Ctn*

Homozygous *arm* null alleles (*arm^2^/arm^2^*) have DC defects characterized by small holes in the dorsal epithelium (Fig. 7C) *arm^2^/arm^2^* larvae also have a fully penetrant cuticle phenotype, characterized by shortened cuticle, severe segment polarity defects (resulting in lawn of denticles replacing well-separated denticle belts), a hole in the head region, and a naked cuticle from the anterior end to the third thoracic segment (Fig. 7C, panel iii) [56], [57]. 90% of *arm^2^/arm^2^*; *Dcas^1^/Dcas^1^* flies had smaller cuticles than *arm^2^/arm^2^* (Fig. 7C, panel iv). Among these, 82% completely lacked cuticle in the head region, and 65% had enhanced posterior curvature suggesting a strong GBR defect (Fig. 7C, panel v, vi). While 40% of *arm^2^/arm^2^*; *Dcas^1^/Dcas^1^* flies retained the lawn of denticles characteristic of an *arm^2^/arm^2^* mutant (compare Fig. 7C, panels iv and vi), 34% had complete deletion of ventral and dorsal denticle belts (Fig. 7C, panel vii), while 26% had well-separated posterior denticle belts (Fig. 7C, panel viii).

We extended our analysis to two additional alleles of arm (*arm^3^* and *arm^8^*). Like *arm^2^*, these alleles express a truncated form of Armadillo [58], [59], due to either amino acid replacements resulting in a stop codon (*arm^2^* and *arm^3^*) or p-element insertion (*arm^8^*) within the arm repeats. *arm^3^* is embryonic lethal typically at stage 16, and is characterized by segment polarity and DC defects, while *arm^8^* undergoes normal DC, but has strong segment polarity defects and dies in pupae. *Dcas^1^* genetically interacts with both *arm^3^* and *arm^8^*, as only 40% of the expected adult progeny with the genotypes *arm^3^/FM7i-GFP*, *B*; *Dcas^1^/Dcas^1^* and *arm^8^/FM7i-GFP*, *B;* *Dcas^1^/Dcas^1^* can be recovered (Table 2).

The p120-catenin homozygotes are viable and fertile; *p120ctn^308^* mutation has been reported to induce a delayed, but complete DC and subtle irregularities of the leading edge in the majority of mutant embryos [60]. A *p120ctn^308^*/*p120ctn^308^*; *Dcas^1^/Dcas^1^* genotype caused misalignment of segments and fused denticle belts (Fig. 7D, right panel, arrow), phenotypes not observed in *p120ctn^308^*/*p120ctn^308^* mutants (Fig. 7D). Double mutant embryos *p120ctn^308^*/*p120ctn^308^*; *Dcas^1^/Dcas^1^* successfully complete embryogenesis and form larvae, but produce few pupae and no adult flies (Figure 7B and Table 2). The later point of lethality may indicate a less direct interaction than that between *Dcas*, *shg*, and *arm*.

### Specificity of *Dcas* genetic interactions

To rule out the possibility of secondary hits accumulated during double balancing and multiple crosses influencing phenotypes, we chose strongest *shg* and *arm* alleles (*shg^2^*, *shg^E17B^ and arm^2^*) double balanced with *Dcas^1^* (*shg^2^/CyO*;*Dcas^1^*/TM6B,*Ubx,y+,shg* *^E17B^* */CyO*; *Dcas^1^*/TM6B,*Ubx,y+,arm^2^/FM7i-GFP,B*; *Dcas^1^*/TM6B,*Ant* *^Hu^* *,y+*) and crossed to Df(3L)Exel6083/TM6B,*Ubx,y+*. No progeny with Dcas^1^/Df(3L)Exel6083 emerged from these crosses, indicating that *Dcas* is indeed indispensible for survival of *shg* and *arm* mutants. When same double balanced flies were crossed to *Dcas^P1^*, less than 50% of *shg* *^E17B^* */CyO*;*Dcas^1^/Dcas^P1^ or shg* *^E17B^* */CyO*;*Dcas^1^/Dcas^P1^* adults expected from the cross emerged (data not shown), indicating sensitivity of *shg* mutants to even slight loss of *Dcas* expression.

Finally, we also explored other potential *Dcas* genetic interactions suggested from studies of the mammalian Cas paralogs NEDD9 and BCAR1. In mammals, NEDD9 interacts with Aurora-A kinase to regulate cell cycle [61], while BCAR1 interacts with the adaptor protein NCK in growth factor signaling [62]. Combination of *Dcas^1^* with mutants in the Drosophila ortholog of Aurora-A (the amorphic alleles *Aur^1^ or Aur^87Ac-3^*) or of Nck (*dreadlocks*, the amorphic *dock^04723^* allele) resulted in no synthetic lethality (Table S1). These negative results suggest a more ancestral and specific relationship of *Dcas* with the other genes yielding positive phenotypes.

## Discussion

This work identifies a strong interaction between the Dcas, and integrin pathway genes, including integrins and their effector kinases Fak56D and Src42A, during early embryonal development in *Drosophila*. The synthetic lethal phenotypes found in double mutants of *Dcas* and *Src* or *FAK56D* were marked by defects in dorsal closure and in some cases by the appearance of anterior cuticle holes that suggested head involution defects. These defects were commonly accompanied by abnormalities in epithelial function, including failure to appropriately localize *shg*/E-cadherin to cell junctions, and reduced *shg* expression. Our data are compatible with the idea that either Fak56D or Dcas is sufficient to support *shg*/E-cadherin localization and cell polarization during morphogenetic movements in *Drosophila* embryos, but the absence of both cannot be sustained.

Building from these observations, we established a novel synthetic lethal relationship between *DCas*, *shg*, and *arm*. As with crosses to alleles of *Fak56D* and *Src42A*, the point of lethality was at the time of dorsal closure, at embryonal stages 15–16, and associated with defective cuticle formation. One way to integrate these observations is to hypothesize that the DCas, Fak56D, and Shg protein products are normally in dynamic balance, with Dcas regulating Shg cycling. The fact that *Crb* and *Dlg1*, a mammalian homolog of *Dlg*, have been reported to support Shg localization to adherens junctions [51], [63], suggests that *Dcas*/*Fak56/Src42A* specifically interact to support this cell polarity/cell junctional control system. In this context, it is suggestive that the Crb family protein CRB3 has been described as part of a complex including CRB3, Pals1, and PatJ that becomes tightly associated with Src kinase during reorganization of cell polarity [64]. In the absence of *DCas* and *Fak56D*, Shg cannot localize properly; the moderately elevated levels of Shg proteins found in these embryos most likely arises as part of a cellular compensatory mechanism in response to decreased functional Shg signaling complexes. In further indirect support of the idea that this is a specific Dcas action, the fact that genetic interactions were not observed between *Dcas^1^* and *Aur* or *Dock* indicates that *Dcas* does not promiscuously interact with other genetic lesions to reduce viability.

A previous study demonstrated a role for Dcas in axonal guidance in the development of the nervous system of adult flies [40]. That work analyzed the hypomorphic *Dcas* mutant allele *Dcas^P1^*, and the small deficiency *Df(3L)Exel6083*, including *Dcas* and five adjacent genes, which we have also used in this study. The earlier study focused exclusively on analyzing the contribution of *Dcas* to axonal guidance in late (stage 16/17) embryos: in that analysis, *Dcas* functioned similarly to integrins, and genetically interacted with integrins (*if*, *mew*, and *mys*) in regulating axon guidance and axonemal defasciculation. In this context, it is intriguing that the mammalian Cas family *NEDD9* gene is abundant during neuronal development, has been proposed as a candidate locus for oral cleft defects in humans based on its chromosomal location near the OFC-1 locus [65], and has recently been implicated in control of neural migration and neuronal cell fate [66], [67]. Together these findings raise the possibility that this specific *Dcas* paralog has a specific role in human neuronal migration and morphogenesis of the head. As with our data using the new *Dcas^1^* allele, homozygous deletion of *Dcas* in conjunction with integrins had moderate effect on viability of adult flies, although our work for the first time demonstrates an interaction between *Dcas* and *if* and *mew*, and also between *Dcas* and *Src*, in regulation of wing development.

Generation of the first null allele of *Dcas* provides a useful new tool to study the role of this protein in *Drosophila* development. This work illuminates the evolutionary conservation of Dcas function within the integrin and receptor tyrosine kinase network, including FAK, Src, and integrins genes. The finding that a low percentage of embryos with mutant *Dcas* and all embryos with double mutations in *Dcas* and *Fak56D*, have perturbed localization of polarity markers, including Shg, indicates a novel function for Cas family in regulation of cell polarity. To date, the evidence directly connecting Cas proteins to a known mechanism for control of cell polarity is sparse. Although NEDD9 was in fact discovered in a functional genomics screen for cell cycle and polarity modifiers in budding yeast (leading to its designation as HEF1, Human Enhancer of Filamentation 1) [5], the mechanism involved was not established, and given the great evolutionary distance involved, may not be relevant to a role in metazoans. Both BCAR1 and NEDD9 interact physically with proteins that influence cell polarity controls during pseudopod extension and other actin polarization processes: these include the GTP exchange factor AND-34 [68] and Rac1 [69].

Our data in the present study indicating genetic interactions with cell-cell junction regulatory proteins Shg, Arm and p120-catenin may have considerable significance in the sphere of cancer research, as it implies that overexpression of Cas proteins may promote cancer progression by influencing the polarized movement of cells and influencing lateral attachments. The fact that one report has indicated interactions between BCAR1 and nephrocystins at cell-cell junctions in polarized epithelial cells [70] implies that a potentially direct interaction of Cas proteins in these structures is conserved through mammals. However, given the known interactions of Cas proteins with FAK and SRC at focal adhesions, another possibility is that Cas may additionally or alternatively impact Shg function through indirect signaling emanating from these structures. Notably, Bui et al. recently reported that NEDD9 overexpression induced by dioxin caused downregulation of E-cadherin [37], and it will be of great interest to study the consequences of overexpressing *Dcas* on *Drosophila* development. Consequences for loss of NEDD9 expression on E-cadherin expression or localization are not yet known. Resolving these questions will provide intriguing directions for future studies.

## Materials and Methods

### *D. melanogaster* stocks, crosses

The following mutant Drosophila stocks were obtained at Bloomington Stock Center and are described in Flybase (http://flybase.bio.indiana.edu/): *shg^2^*, *shg^K03401^*, *shg^E17B^ arm^2^*, *arm^3^*, *arm^8^*, *Src42A^10108k^*, *Src42A^JP45^*, *Src42A^E1^*, *src42A^miri^*, *aur^1^*, *aur^87Ac-3^*, *dock^04723^ and p120ctn^308^*, *mew^EY09631^*, *If^B2^*, *If^3^*, *mew^G0429^*, *mys^1^*. The *fak56D^CG1^* strain was obtained from Ruth H. Palmer (Umea University, Sweden). The stock containing the *Dcas^P1^* allele was provided by Dr. Kolodkin (Johns Hopkins School of Medicine, Baltimore). Green double balancer Fm7i, B, Kr::GFP/+; ki/TM6B, Tb, Kr::GFP was used for double balancing Dcas and genes located on X chromosome. Balancer stocks Tm3, Sb'/Tm6, Dr', w, TM2, Ubx'/Tm6, Sb' and yw, Sco/CyO; ki/TM6B, Ubx, y^+^ and green compound double-balancer w^1^; T(2;3)CyO-TM3, P{GAL4-Hsp70.PB}TR1, P{UAS-GFP.Y}TR1; P{GAL4-Hsp70.PB}TR2, P{UAS-GFP.Y} TR2, y^+^ Ser^1^/noc^Sco^; Sb^1^ stocks from Bloomington Stock Center were used to balance the null *Dcas^1^* mutation. To create double balanced *If*, *mys* and *mew* and Dcas stocks, we used the double balancer *FM7i*, *B*, *Kr::GFP*; *ki/TM6B*, *Tb*, *Kr::GFP*, which constitutively expresses GFP on chromosomes X and III. FAK56D, Src42A and other chromosome II mutants were double balanced with *yw*; *Elp/CyO*; *ki/TM6B*, *Ubx*, *y+* green balancer. Double balanced mutant alleles were then crossed to double-balanced *Dcas* alleles, i.e *Dcas^1^*. We also attempted to generate double mutants in Dcas and Src64B. Unfortunately, the very close location of the *Dcas* and *Src64B* loci prevented successful recombination involving these mutations.

### Construction of CG1212/Dcas knockout and viability calculations

To generate a *Dcas* null allele, yw hs-flp; FRT82neurIF63/Tm3, Sb' females were crossed to FRT-containing PBac{WH}f00059 (BDSC, Bloomington, IN) males to produce yw hs-flp/Y; PBac{WH}f00059/Tm3, Sb' males, which were backcrossed to yw hs-flp; FRT82neurIF63/Tm3, Sb'. Next, yw hs-flp; PBac{WH}f00059/Tm3, Sb' females were crossed with w, P5-HA-2428 males (Szeged Drosophila Stock Centre, Hungary). Male progeny with the genotype yw hs-flp/Y; PBac{WH}f00059/P5-HA-2428 were backcrossed to yw hs-flp; PBac{WH}f00059/Tm3, Sb' to obtain yw hs-flp; PBac{WH}f00059/P{RS5}5-HA-2428 flies. We then initiated double stranded breaks and isolated knockouts as described in [71]. Endpoints of excision were defined by the P-element P{RS5}5-HA-2428, positioned within 50bp from the Dcas start codon and containing an FRT site in the same orientation as the piggyBac Pbac{WH}f00059 transposon, located in between the Dcas ORF and the adjacent downstream gene CG7049 (Fig. 1A). Flippase-activated excision produced 20 potential mutant stocks, which were then analyzed by quantitative RT-PCR with seven sets of primers spanning the Dcas coding region, and in flanking genes (Fig. 1A). Correct endpoints of the *Dcas* deletion were initially confirmed using qRT-PCR to analyze DNA from adult flies, using primers directed at the promoter, and first and last coding exons of *Dcas*, as well as flanking upstream and downstream chromosomal sequences, to confirm that the *Dcas* gene was not detectable in *Dcas^1^/Dcas^1^* mutant stocks, although readily detected in WT flies. *Dcas* transcript levels were also measured in knockout flies using qRT-PCR to analyze at least 3 independent samples of RNA prepared using the RNeasy kit (Qiagen) from adult flies and larvae. *Dcas^1^* null flies were serially backcrossed to 3^rd^ chromosome balancer stocks (TM3,TM6 and TM2) to exclude additional recombination-associated mutations on other chromosomes before further characterization.

Double balanced heterozygous adults containing mutations in Dcas and prospective interacting genes were selected and crossed together to establish stocks and to assess viability of adult progeny. The total number of expected progeny was calculated from the number of phenotypically viable double balanced adult heterozygotes, i.e. *Fak56^CG1^/CyO*; *Dcas^1^/*TM6B, *Ubx*, *y+*, which were considered 100% viable. The percentage of viability for the rest of the progeny was calculated in agreement with Mendel's law of independent assortment for two alleles, which was also used to calculate the ratio between different genotypes in the progeny. If both mutant alleles were viable, i.e. *Fak56^CG1^* and *Dcas^1^*, the ratios were as follows: *Fak56^CG1^/CyO*; *Dcas^1^*/TM6B, *Ubx*, *y+*: *Fak56^CG1^/Fak56^CG1^*; *Dcas^1^*/TM6B, *Ubx*, *y+*: *Fak56^CG1^/CyO*; *Dcas^1^/Dcas^1^*: *Fak56^CG1^/Fak56^CG1^*; *Dcas^1^/Dcas^1^* as 4∶2∶2∶1. If one of mutants alleles was lethal, i.e. in a combination of *Src^K10108^* and *Dcas^1^*, the *Src^K10108^/CyO*; *Dcas^1^*/TM6B, *Ubx*, *y+*: *Src^K10108^/CyO*; *Dcas^1^/Dcas^1^* ratio was 2∶1. Progeny from more than three independent crosses was collected and represented as tables modeled after [46].

### Preparation of embryos and immunohistochemistry

For analysis of localization of polarity markers, embryos were prepared as described by [72], with minor modifications. Briefly, embryos collected off apple-agar plates were washed in 50% bleach for 2 minutes, then rinsed twice in PBS and gently shaken on a platform for 40 minutes in 1∶1 8% PFA/heptane mixture containing 2 units/ml of phalloidin (Invitrogen, Carlsbad, CA). Embryos accumulating at the interface between PFA and heptane were collected, applied to double stick tape, then de-vitellinated with a fine glass needle. Rehydrated embryos were blocked with 10% BSA/PBS for an hour, washed in 1% BSA/PBS, and incubated with a primary antibody for 2 hours, followed by two 5-minute washes in PBS, and incubation with secondary antibody for an hour. Embryos were visualized with Leica TCS SL and Nikon C1 confocal microscopes, and images analyzed using Metamorph and EZ-C1 freeViewer software.

### Western blot analysis

Whole embryo, larval and fly lysates were prepared by homogenization in 3× Laemmli sample buffer containing 10% SDS, and then boiling for 5 minutes. Samples were separated by 10% Bis-Tris NuPage PAGE (Invitrogen, Carlsbad, CA). Western blots were performed using standard protocols.

### Antibodies and visualization reagents

Primary antibodies used included ECCD-2 mouse α-E-cadherin (Invitrogen, Carlsbad, CA), rat E-cadherin, mouse α-Fasciclin3, rabbit α-crumbs (DSHB, Iowa), α-zeta-PKC and α-Dlg (Santa Cruz Biotechnology, Inc, Santa Cruz, CA), mouse HRP-conjugated β-actin (Abcam, Cambridge, MA). Secondary antibodies included HRP-conjugated α-mouse, -rat or –rabbit (Amersham, Pittsburgh, PA) or Alexa-fluor-488, -568 or -633-conjugated α-mouse, -rat or –rabbit ((Invitrogen, Carlsbad, CA). Nuclei were stained with DRAQ5 reagent (Cell Signaling, Boston, MA).

### Cuticle preparation

Embryos were collected for 26 hours after the parents were removed from the apple-agar plates, dechorionated with 50% bleach, devitellinized in 1∶1 heptane/methanol for 15 minutes, washed 2× with methanol, 2× with lactic acid, transferred on a slide into a drop of Hoyer's medium, and photographed using a phase contrast microscope (Nikon Eclipse TE-2000-U). The stocks carrying mutations in a gene of interest and Dcas were rebalanced over a green compound balancer to separate GFP-negative double homozygotes and GFP-positive double heterozygotes. Homozygotes were then collected for cuticle preparations. Where applicable, GFP-negative homozygotes and GFP-positive heterozygotes were separated using fluorescent dissecting microscope, prior to bleaching.

### Wing preparation

Wings were detached and mounted into Hoyer's medium. Images were taken at 4× magnification on Leica TCS SL microscope.

### Embryo Hatch Rate Analysis

Mutant embryos were collected from indicated crosses. For hatch rate determination, embryos were collected on apple-agar plates for 4 hr, than the parents were removed and more than 500 embryos per genotype were counted after 2 days' incubation.

### Real Time PCR analysis

Stage 13–15 embryos were bleached and rinsed with distilled water. 30 mg of embryos were used to isolate mRNA with the RNAeasy kit (Qiagen, Valencia, CA) and overall mRNA quality assessed using an Agilent Bioanalyzer (Agilent, Santa Clara, CA). The concentration of *Shg* mRNA in was quantified via real-time PCR with the Smartcycler detection system (Cepheid, Sunnyvale, CA) using SYBR green I (Molecular Probe, Eugene, OR) in three independent experiments. The primers used in the PCR reaction were as follows: Shotgun, forward primer 5′- GCGCTACGACGAATCCATG-3′ and reverse primer 5′- AGATAATACCCGACTCCTTGTCAATC-3′; and as a normalization control, the housekeeping gene RpII140, forward primer 5′-CGCACGTGGAAGTTGGTAAT-3′ and reverse primer 5′- ACAATCAGAGTCCGCGTA ACAC-3′.

## Supporting Information

Table S1 Additional genetic interactions of Dcas^1^. For data shown, the parental crosses for Dcas^1^ and dock^04723^ were performed as described in Materials and Methods. Alleles of Aurora kinase (aur^1^ and aur^87Ac-3^) were first recombined to position both mutations to the same chromosome with Dcas^1^, balanced over TM3, Ser balancer to establish a double heterozygous stock (i.e. aur^1^, Dcas^1^/TM3). Double heterozygotes were crossed with Dcas^1^/Dcas^1^ to produce aur^1^, Dcas^1^/Dcas^1^, aur^87Ac-3^, and Dcas^1^/Dcas^1^ which were then crossed back to double heterozygotes. The viable progeny of indicated genotypes was collected and compared to phenotypically normal double heterozygous siblings, (i.e. aur^1^, Dcas^1^/TM3) in each of 3 independent experiments.
(0.03 MB DOC)
