# Supplementary material for: Dcas Supports Cell Polarization and Cell-Cell Adhesion Complexes in Development
Source: PLoS One. 2010 Aug 24;5(8):e12369. doi: 10.1371/journal.pone.0012369 (PMC2927436; doi:10.1371/journal.pone.0012369)
Supplement: Table S1 — Additional genetic interactions of Dcas1. For data shown, the parental crosses for Dcas1 and dock04723 were performed as described in Materials and Methods. Alleles of Aurora kinase (aur1 and aur87Ac-3) were first recombined to position both mutations to the same chromosome with Dcas1, balanced over TM3, Ser balancer to establish a double heterozygous stock (i.e. aur1, Dcas1/TM3). Double heterozygotes were crossed with Dcas1/Dcas1 to produce aur1, Dcas1/Dcas1, aur87Ac-3, and Dcas1/Dcas1 which were then crossed back to double heterozygotes. The viable progeny of indicated genotypes was collected and compared to phenotypically normal double heterozygous siblings, (i.e. aur1, Dcas1/TM3) in each of 3 independent experiments. (0.03 MB DOC) [file pone.0012369.s001.doc]

**Supplementary Table S1**. **Additional genetic interactions of *Dcas^1^*.** For data shown, the parental crosses for *Dcas^1^* and *dock^04723^* were performed as described in Methods. Alleles of Aurora kinase (*aur^1^*and *aur^87Ac-3^*) were first recombined to position both mutations to the same chromosome with *Dcas^1^*, balanced over TM3, Ser balancer to establish a double heterozygous stock (i.e. *aur^1^, Dcas^1^/*TM3). Double heterozygotes were crossed with *Dcas^1^/Dcas^1^* to produce *aur^1^, Dcas^1^/Dcas^1^*, *aur^87Ac-3^,* and *Dcas^1^/Dcas^1^* which were then crossed back to double heterozygotes. The viable progeny of indicated genotypes was collected and compared to phenotypically normal double heterozygous siblings, (i.e. *aur^1^, Dcas^1^/*TM3) in each of 3 independent experiments.

| **Crosses** | **Genotype of mutant progeny** | **Viability (+/-SD) (%)** | **Total (n)** |
| --- | --- | --- | --- |
| ***dock^04723^/Cyo; Dcas^1^/* TM6B *x dock^04723^/Cyo; Dcas^1^/* TM6B** | ***dock^04723^/Cyo; Dcas^1^/ Dcas^1^*** | **108 (+/-20)** | **1402** |
| ***aur^1^, Dcas^1^/*TM6B *x***  ***aur^1^, Dcas^1^/Dcas^1^*** | ***aur^1^, Dcas^1^/Dcas^1^*** | **101 (+/-4)** | **742** |
| ***aur^87Ac-3^ , Dcas^1^/* TM3 *x aur^87Ac-3^ , Dcas^1^/ Dcas^1^*** | ***aur^87Ac-3^ , Dcas^1^/ Dcas^1^*** | **96 (+/-3)** | **890** |
